# Exploration of the integration of care for persons with a traumatic brain injury using social network analysis methodology

**DOI:** 10.5334/ijic.1055

**Published:** 2013-11-04

**Authors:** Marie-Eve Lamontagne

**Affiliations:** Department of Rehabilitation, Université Laval, 2325 Rue de l'Université, Quebec City, QC G1V 0A6, Canada

**Keywords:** traumatic brain injury, brain injury, health care system, community network, social network analysis, social network, evaluation

## Abstract

**Introduction:**

Integration is a popular strategy to increase the quality of care within systems of care. However, there is no common language, approach or tool allowing for a valid description, comparison and evaluation of integrated care. Social network analysis could be a viable methodology to provide an objective picture of integrated networks.

**Goal of the article:**

To illustrate social network analysis use in the context of systems of care for traumatic brain injury.

**Method:**

We surveyed members of a network using a validated questionnaire to determine the links between them. We determined the density, centrality, multiplexity, and quality of the links reported.

**Results:**

The network was described as moderately dense (0.6), the most prevalent link was knowledge, and four organisation members of a consortium were central to the network. Social network analysis allowed us to create a graphic representation of the network.

**Conclusion:**

Social network analysis is a useful methodology to objectively characterise integrated networks.

## Introduction

In recent decades, the development of integrated care for various populations has become an important topic of discussion among clinicians, managers, policy-makers and scholars in many countries. However, one of the few consensual opinions about integration of care is that it represents, in fact, a very versatile reality that can take various forms. As an example, Contandriopoulos, Denis, Touati and Rodriguez [1] broadly define integration as a process that consists of creating and maintaining over time a common governance between independent stakeholders and/or organisations with the goal of coordinating their interdependencies to allow them to cooperate in achieving a common objective. This definition recognises that integration can be implemented at many levels within health care systems, such as between the clinicians of a given team, between the teams of an organisation or between the organisations in a given area. Integration of various entities (individuals, teams or organisations) using various ties (communication, collaboration, information exchange, etc.) creates distinct realities that can be conceptualised as networks. Indeed, since a network is broadly defined as a set of nodes (constituencies) related by various types of ties (relationships) [2,3], it is possible to identify the concept in many experiences and innovations in the health care field as in other fields such as strategic management, human behaviour study, sociology, etc. [4,5]. Thus, networks may be created at the micro-level, by or around one individual, they might happen in small groups or communities at the meso-level, or even in large societal settings such as health care or social care settings.

Some authors have suggested that the polyvalence of a network has created confusion about the concept, leading to the lack of a unique or standard network terminology. Among other consequences, this confusion makes it difficult to study and compare networks [6]. Some tools and strategies have been proposed [7–9] to describe and measure integration; however, none of them have been widely used nor have they been used through the various levels of a health care system.

Given the popularity and complexity of integrated forms, it is increasingly relevant to expand the knowledge base about networks. Indeed, it is important to document which kinds of integrated forms would best help meet people's needs, which are the most cost-effective, and which are the most suitable in a given context. The study of networks and integrated care needs must rely on a systematic, valid and reliable methodology that will allow individuals interested in this topic to describe, measure and compare the integrated forms that they are interested in.

A well-documented methodology already exists that can be used to characterise networks and study service integration [10]. Social network analysis, a methodology dedicated to the study of network structures [10], has been used for decades in sociology [11], political science and public management [4] to understand how individuals and organisations work together. As such, this methodology is highly pertinent to the study of integrated care. In fact, the use of social network analysis methodology has been described as a key analytical approach to the study of integrated care [12]. Social network analysis involves collecting and analysing data from multiple individuals or organisations that may be interacting with one another [13]. The methodology's unique contribution is to focus on the *relationships* between individuals or organisations [13,14] and not on the individual/organisation itself. Social network analysis methodology has already been used to investigate patterns of health care delivery such as referral patterns, service integration, coordination and collaboration [10]. There are many excellent texts with in-depth descriptions of the essential concepts of social network analysis [2–5,8,10,11,13,15–19], but to our knowledge, social network analysis has rarely been used to describe and measure the integration of health and social care in neurotrauma systems. The goal of this article is to illustrate social network analysis use in the context of integrated systems of care for individuals with traumatic brain injury.

## Method

Social network analysis enables the measurement of the characteristics of (1) the network itself, (2) the network participants and (3) links that connect the participants within the network. Specific measures (e.g. density, centrality, multiplexity, etc.) or diagrams (often called sociograms) provide a relatively objective snapshot or measure of the network at a given time [20].

We performed a social network analysis from September 2009 to January 2010 in the context of a single-case study [21] of integrated systems of care for individuals with traumatic brain injury in the province of Québec, Canada. In Québec, a general trauma network exists for any individual who sustains a serious traumatic injury (orthopaedic trauma, spinal cord injury, traumatic amputation, serious burns). The network encompasses organisations offering emergency care (911 dispatchers and dispatch centres, police and first responders, ambulance attendants), hospital services (stabilisation, primary, regional secondary, secondary and tertiary care) and post-hospital services (rehabilitation, community maintenance). While the organisation members of the network may be clearly identified, the relationships between most of them are not very formalised, with some notable exceptions. For example, there are some protocols regulating the transfer of trauma patients between hospitals offering various levels of services, and ambulance companies usually have contracts with the regional health authority.

In addition, embedded within the general trauma network are eight formalised traumatic brain injury sub-networks or consortiums, each covering different sociodemographic regions. Consortiums represent a small part of the trauma network and include facilities specifically designated to provide care to persons who sustain a traumatic brain injury. Each consortium minimally includes five organisations or a trauma centre, a rehabilitation facility offering in-patient rehabilitation, a rehabilitation facility offering out-patient rehabilitation and the regional health authority working in close collaboration with the traumatic brain injury consumer association in the region. Consortium members have to work closely together to reinforce their links to ensure coordination between hospitals, rehabilitation facilities and other organisations providing physical health care, rehabilitation and support services to persons with traumatic brain injury. Consortium members are jointly responsible for establishing the continuum of care, providing care to their clients, and coordinating services at the network level. Consequently, members have to establish links with other organisations that are members of the trauma network outside of their consortium. This creates a complex, two-step service delivery model involving many actors related by links that vary in type and quality. Social network analysis thus seems to be an ideal methodology to study the characteristics of traumatic brain injury consortiums in Québec.

The network being studied was chosen, because it was considered to be a productive network by three individual experts from the provincial advisory board responsible for evaluating and accrediting traumatic brain injury consortiums. Located in a semi-urban area of 474,000 inhabitants (11.1 individuals/km^2^), it covers a geographical area of 43,000 km^2^ (3% of the total area of the province). The consortium embedded in the provincial trauma network was created in 2000 and was made up of four organisations (rather than five) since the rehabilitation centre offers both in- and out-patient rehabilitation services. Ethics approval was obtained from the Centre for Interdisciplinary Research in Rehabilitation of Greater Montreal prior to data collection.

## Participants

Identifying participating network organisations can be a challenging task in social network analysis since network boundaries are seldom clearly defined [10,13]. In our study, we used positional and relational strategies (Appendix 1) to identify potential network participant organisations. First, based on the minutes of the consortium's meetings between 2001 and 2009, we listed all the organisations the consortium had been in touch with in one way or another during this period (a positional strategy). Then, using relational strategies, we asked the representatives from the consortium to make their own list of organisations they felt were their network's partners, and to provide contact information for key informants at each of those organisations. The results of these two strategies were combined to create a preliminary network member list. The list was revised with the help of an expert from the government-appointed advisory board that evaluates trauma networks in Québec, and a few other potential organisation members were added. The combination of the two strategies resulted in a final list of 43 potential participating organisations: 11 (26%) ambulance companies, eight (19%) acute care hospitals designated as trauma centres, six (14%) stand-alone rehabilitation facilities providing in- or out-patient rehabilitation services, nine (21%) community-based organisations including health and social services centres, and nine (21%) other organisations (e.g. regional health authority, automobile insurance board, workers compensation board, provincial government-appointed advisory board, Provincial Health and Social Services Ministry, local and regional trauma committees). The list also contained the names of one key informant for each potential organisation.

## Data collection

We used a survey technique [22] to gather network data. All potential network members were contacted by phone, and the goals of the study were explained to them. If they agreed to participate, these individuals were asked to provide a valid email address so that a link to a web-based questionnaire (described below) could be sent to them, along with a consent form and sociodemographic questionnaire. Participants were given one month to complete the questionnaire, after which email reminders were sent.

SurveyMonkey, a web-based platform, was used to make the survey available to the participants over the Internet. The network data collection tool proposed by Provan and colleagues [13] was adapted to optimise its use online. As suggested by these authors, each participant was provided with a complete list of potential network partner organisations and asked to indicate whether, to their knowledge, their own organisation shared links with each of them in their provision of services to persons with traumatic brain injury. The participant was then asked to characterise the type(s) and quality of the links that existed between organisations. The questionnaire was developed to investigate: (1) Knowledge, (2) Communication, (3) Human resource sharing, (4) Financial resource sharing, (5) Material resource sharing, (6) Client referrals and (7) Formalisation of contracts and protocols (see Appendix 2 for the complete description of the links investigated). Participants were invited to add and rate any other link(s) that their organisation had with other organisation(s). Participants indicated ‘0’ if their organisation did not share any other link, or were asked to indicate the quality of each existing relationship by rating it as ‘1’ (poor), ‘2’ (fair), ‘3’ (good) or ‘4’ (excellent).

In our study, the SurveyMonkey platform automatically compiled all of the questionnaire data in an Excel file that was then downloaded. When there was more than one participant per organisation, the data were aggregated in such a way that the highest of the three scores for each organisation and each relationship was used for analysis. This was done because the choice was made to record the most optimistic view of the network. The Excel file was organised into seven distinct matrices, one for each type of relationship, and then imported into UCINET. A binary version of each of the seven matrices was produced and an eighth matrix (summative) was created, based on the total number of relationships existing between all organisations. The summative matrix values ranged from 0 (no relationship) to 7 (all possible relationships).

## Analysis

Many social network analysis measures were used in this study. The ‘scope’ of a network simply consists of the number of network members, while its ‘density’ is a measure of network cohesion [10]. More specifically, density is defined as the total number of relational ties present, divided by the number of possible relational ties. Because it is a ratio, the density value varies between ‘0’ and ‘1’, where ‘0’ indicates a total absence of links in the network and ‘1’ means all possible relationships existing in the network. A frequent measure pertaining to network members is Freeman's ‘degree centrality’, which is an indicator of the prominence of the organisations. Degree centrality consists of the number of ties one finds upon a node through a given type of link. The higher the centrality value, the more influential or important the organisation is with regard to this type of link. When the relationships are directed, centrality can be computed for links directed towards a given network member (in-degree centrality) and for links that are sent out by this organisation (out-degree centrality) [10]. Finally, ‘multiplexity’ is useful in characterising the strength of network relationships. It consists of the number of links existing between a dyad of network members, and it is often used as a proxy for the strength of the links, since a relationship based on a variety of links is less likely to disappear if a given type of link breaks. If such measures are collected, it is also possible to draw conclusions about the quality and frequency of relationships.

## Results

Initially, to gain a better understanding of the links existing at various organisational levels, the team attempted to enrol three participants (a clinician, a coordinator and a manager) from each organisation with the goal of examining the perceived pattern of relationships at each level. However, early in the recruitment process, enrolment was difficult since many individuals from the various organisations either were not even aware that the network existed or had very limited contact with network member organisations. Out of 43 potential organisations, 12 organisations were recruited. Multiple participants within an organisation were only recruited from the 4 organisation members of the consortium. The 12 distinct organisations that took part in the study were: an ambulance company, three hospitals, four rehabilitation centres, one community-based organisation and three ‘other’ organisations. In all, 24 participants (56%) were recruited, and 18 (42%) completed the survey. Non-participants either did not return our repeated calls (70%) or indicated they did not know about the network and could not name someone else in their organisation who might know about it (30%). Three participants were clinicians, seven held coordination positions, while eight held managerial positions. Participants had an average of 16.8 years of experience in their organisation.

**Figure 1.**
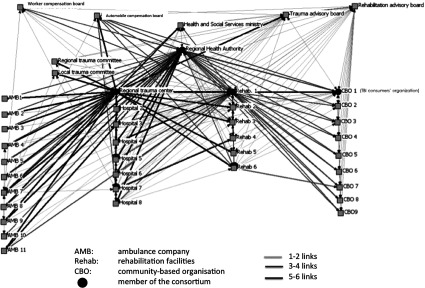
Illustration of the network and consortium.

We first use a graph to visually depict the network (Figure 1). It was decided to exclude the ‘Knowledge’ link, because preliminary analysis showed that almost all the organisations within this small regional network knew about each other; thus, including this type of link in the network was deemed uninformative. Organisation members of the consortium were indicated with a circle, while other organisations in the network were represented with squares. Links represented by the pale lines reflect the presence of only one or two types of relationships between partners, medium lines reflect the presence of three or four links, while bold links mean that partners were linked through five or six types of relationships. The graph shows that the Regional Trauma Centre fosters numerous links with many other organisations, particularly ambulance companies. A Rehabilitation Facility (Rehab 1) and the Regional Health Authority also seem to have several relationships with most of the other organisations. Notably, they are linked, albeit somewhat weakly, with Health and Social Service Centres corresponding to Community-Based Organisations #2 to #9. Community-Based Organisation #1, corresponding to the traumatic brain injury Consumer Association, was also strongly linked to Rehabilitation Facility #1.

Network measures showed that the network studied was moderately dense, with an overall density index of 0.60, implying that, overall, 60% of the potential relationships between organisations actually existed (Table 1). The densest links were based on knowledge and communication, while those related to resource sharing were the weakest. Formalisation of contracts and protocols occurred between 20% of the 43 network organisations, while about one-third of the organisations reported referring clients to one another. In addition, social network analysis showed that relationships were denser between consortium members than between the members of the rest of the network. The density values for each relationship were higher for this subset of the network than for the whole network.

In the general network being studied, the mean centrality value was 11.7 and values ranged from 0 to 41 (a value of 41 indicates that an organisation is linked with 41 others, and a value of 0 indicates that an organisation is unknown to the other network members). While the majority (24 organisations) had a centrality < 10, two organisations had a centrality > 40, two had a centrality > 30, four had a centrality > 20, nine had a centrality > 10, and one had a centrality of 0. The consortium members had the highest degree of overall centrality, with a value of 41 for both the Rehabilitation Centre and the Regional Health Authority, while the Trauma Centre had a centrality of 34 and the traumatic brain injury Consumer Association a centrality of 28. The Advisory Board responsible for the designation of consortiums also had a high centrality value of 31 (Table 2).

Organisations' centrality also varied according to the type of relationship examined. For example, the Regional Health Authority was found to be more central with regard to communication links, while the Regional Trauma Centre was more central with regard to all resource sharing, and the Rehabilitation Centre was better in transferring traumatic brain injury patients to and from other organisations.

The multiplexity of the relationships that exist between each dyad was also determined. Participants described their own organisations as having an average of 1.7 relationships (±2.0) with each other organisation, out of a possible 7, indicating somewhat weak relationships. Only 28% of the links described were confirmed (i.e. described by both partners). The maximum mean multiplexity value was 4.8 (the Regional Trauma Centre), while the lowest value was 0.8 (for one ambulance company and two community health organisations). Hospitals typically established the strongest links (mean multiplexity value of 2.5), while ‘other’ organisations and rehabilitation facilities maintained moderately strong links with others (mean multiplexity values of 1.9 and 1.4, respectively). Ambulance companies and community-based organisations had the weakest links with others (mean multiplexity indexes of 1.2 and 1.1, respectively). Again, consortium members showed a higher level of multiplexity than did the general trauma network members. The multiplexities of the Regional Trauma Centre, Regional Health Agency, Rehabilitation Facility and traumatic brain injury Consumer Association were 4.8, 3.9, 3.0 and 1.8, respectively.

Finally, social network analysis data were used to investigate the quality of the various links. In general, participants graded the links as being Good or Excellent and relatively few links were described as Poor or Fair (Table 3). The formalisation of contracts and protocols appeared to be particularly satisfying, since almost half of them were deemed Excellent and none was considered Poor. With regard to the quality of the links developed by different types of network members, participants reported that 91%, 88% and 87% of the relationships with trauma centres and hospitals, with ‘others’ and with other facilities, respectively, were Good or Excellent. The quality of the links with the ambulance companies and with community-based organisations was lower, since 76% and 58% of the relationships respectively were deemed Good or Excellent. The proportion of links deemed to be Poor or Fair was higher (43%) for health and social service centres than for other types of organisations. In contrast, a higher proportion (76%) of the relationships between consortium members were deemed Excellent, 23% were Very good and only 1% were Poor (data not presented). While knowledge, communication and client referrals were rated uniformly as Excellent, resource-sharing relationships (financial, human and material) were typically rated as lower quality.

## Discussion

The goal of this article was to use a case study to illustrate social network analysis use in the context of integrated care. The network members and researchers found this methodology useful, and it could have been used in other consortiums, trauma networks or traumatic brain injury teams to measure the integration of care in a common and shared way. Thus, our conclusions are similar to those of Provan and Sebastian [23], and they support the assumption that social network analysis could represent a useful, feasible and reproducible methodology that could be key to the study of integrated care. The following discussion focuses on the impact of our methodological choices. The conclusion provides an overall analysis of the opportunities and constraints related to using this method to describe integrated care.

Regarding the importance of delineating network boundaries, multiple strategies were used to identify the 43 potential participating organisations in the network. Following the suggestion of Provan et al. [13], all of these organisations were included in the study ‘…to allow the respondents to determine which organisations are part of the network and which are not’ (p. 606). However, using multiple strategies might have led to the inclusion of representatives of organisations that were not active members of this particular traumatic brain injury care network but who should be. On the one hand, the low participation rate may be because many of the organisations did not feel like they were part of the network. It might also be due, despite our efforts, to inadequate or insufficient reminders due to ethical considerations (i.e. not press participants who changed their minds and did not want to continue to participate in the study). The low participation rate makes it difficult to generalise our results to the whole network, and further measures of this network are required before consistent observations can be made. On the other hand, the over-representation of potential network member organisations may have been caused by a social desirability bias, where participants wanted the researchers to think their network was larger and stronger than it really was. One way to counter this bias would be to include an organisation only if it has been cited as a network member by at least two respondents [18]. This would pose a greater risk of missing occasional but important partners, but it might also decrease the number of non-respondents. Since both formal and informal links were being investigated, the mix of positional and relational bounding strategies may have been optimal. Using only a positional strategy would have led to the description of the ‘formal’ network and the potential omission of network members such as community-based associations that do not have a formal or recognised status within the healthcare system. In contrast, using only a relational strategy would have reduced the probability of including organisations recognised by the Health Ministry as official members of the traumatic brain injury care network but that rarely participate in it.

In the context of the present case study, we chose to gather the social network analysis data using a survey since it seemed to be the best way to collect data from network participants scattered over a large geographical area. However, the lack of direct interactions between researchers and potential participants may have decreased participants' motivation to complete the questionnaire, thus contributing to the lower than expected participation rate. The survey questionnaire sought to document the quality of seven distinct types of relationships likely to occur between traumatic brain injury care network members. The research team determined that rating seven types of links rather than simply the presence or absence of undetermined relationships would enable participants to provide a more precise and discriminating picture of their networks. Indeed, many types of relationships may co-exist between various dyads of network members, and each of these relationships could be different. Moreover, the use of a Likert scale to rate the quality of the relationships rather than just their binary presence/absence also added to the sensitivity of the network measures. This aspect may have added to the respondents' burden and to the complexity of the analyses. However, we believe this study achieved a good balance between specificity and practicality issues.

In analysing the data, constructing the matrix was greatly facilitated by the use of a web-based platform which automatically transferred survey data into Excel files. Symmetrical data were generated to deal with the different relationship ratings as well as with non-respondents. This choice simplified the analyses and allowed for the presentation of succinct summary results without compromising the overall network picture. It also provided a way to handle non-respondents by assigning the same grades to them as the ones provided by their partner dyad member. However, by using this method, some information was lost regarding the direction of relationships, such as which organisations send clients to the others.

In this study, it was decided to make the data symmetrical by using the maximum quality value. Choosing this procedure provides an optimistic view of the network, while choosing the minimum quality value would have produced a more conservative view. Since the rating scale used was categorical, it did not appear appropriate to compute the mean or median values from the categories. Since the goal of this case study was to describe the traumatic brain injury care network and empower network members to increase their knowledge about their organisation, it was determined that using a conservative picture of the network might have a discouraging effect on the network members while using the most optimistic picture might more easily sustain discussions. In addition, it might have enabled the sharing of visions and ideas for improvement, which could be useful to network functioning and development.

Social network analysis has often been criticised as being only a ‘descriptive’ methodology [11]. However, description and measures of networks are instrumental in better appraising their characteristics and explaining variations in network implementation, functioning and performance [18]. Indeed, social network analysis outcomes can be used for scientific purposes to increase our understanding of the contribution of network characteristics to outcomes such as performance, effectiveness, cost, satisfaction, quality of life, etc. Social network analysis can be used to longitudinally track the implementation and progression of a network. Social network analysis outcomes can also be useful to compare networks. Social network analysis has been used in Canada to examine the effectiveness of community-based dementia care networks [24] and in the United States to design quality improvement teams [25], to study the effectiveness of community-based mental health networks [26] and to assess the provision of chronic disease services [20]. Social network analysis can also be used by networks and communities in the field to help them understand and assess their own partnerships and networks [13,20]. Indeed, Provan et al. [13] describe social network analysis as an objective and systematic tool to help network members reach a shared vision of their whole network, which counterbalances a vision conditioned by organisation members' perspectives and preoccupations. Network members can thus conduct a social network analysis and use it to highlight strengths, weaknesses and areas for improvement within their network.

This case study was essentially descriptive, since initially, there was no clear picture of what a traumatic brain injury care network might look like. Social network analysis was used along with other data collection tools to gain a deeper understanding of this network and to understand how the consortium is embedded within the larger trauma network. During a knowledge translation meeting, the social network analysis outcomes were presented to the consortium members, who found them very interesting. They showed appreciation for the way social network analysis provided a clear snapshot of their collaboration and of the advantages of being in a formalised consortium. They commented on how they needed to increase ‘the number of bold lines and circle nodes’ (link multiplexity and organisation centrality). The consortium members also decided to use social network analysis to measure the success of one of their projects aiming to increase the number of links with community-based organisations. It is thus likely that in the future, social network analysis will be embedded in routine network operations to help members monitor the status of their traumatic brain injury care network.

Overall, we found social network analysis to be useful in providing a reasonably detailed descriptive snapshot of a traumatic brain injury care network. However imperfect, this picture of the network will allow network members to reflect upon their actual network configuration and links and to improve their network activities. If social network analysis had been used to describe other existing networks and consortiums, using the identical methodology would have allowed a comparison between the Québec-based network and the U.S. Defence and Veteran Network, U.K. Rehabilitation Services and the Toronto Acquired Brain Injury Network. Similarities and differences could have been identified in terms of network member types and centrality and the type of links involved.

## Conclusion

This study's main contribution was to provide traumatic brain injury practitioners, managers and researchers with a valid and systematic tool to measure the network structure for traumatic brain injury care. From this perspective, it illustrates the potential contribution that social network analysis could have in opening up the ‘black box’ of integrated care organisation and delivery. Given the rising popularity of integration as a way to improve uncoordinated and fragmented health systems, social network analysis could become a useful method of providing a snapshot of the existing levels of integration within a network and thus help organisation partners to develop a shared view of their network. However, it is important to remember that the current social network analysis was cross-sectional and conducted in the context of a single-case study. Thus, the picture of this particular network cannot be generalised to other traumatic brain injury care networks in Canada or Québec, and it might not reflect the configuration of the network in the following months or years. In terms of study limitations, difficulties were encountered in delineating the boundaries of the network and this probably lowered the participation rate. Some of the methodological choices (making data symmetrical, use of unconfirmed links, use of optimistic results) may have influenced the interpretation of the findings. At the outset, it is important that researchers and network members make informed methodological choices about social network analysis and continue to discuss the strengths, weaknesses, opportunities and constraints that this methodology presents in investigating traumatic brain injury care networks.

If integrated care networks are truly a way to increase continuity at various levels of health systems, it is vital to be able to relate their structures to the performance of the health care system. By providing a tool to characterise inter-organisational networks of care, social network analysis helps to accomplish the first step in this process.

## Reviewers

**Frances Cunningham**, Senior Research Fellow, Menzies School of Health Research, Brisbane, Australia.

**Jean-Pascal Devailly**, MD, Service de Médecine Physique et de Réadaptation, Hôpital Bichat Claude Bernard - Hôpitaux Universitaires Paris, France.

One anonymous reviewer.

## Figures and Tables

**Table 1.**
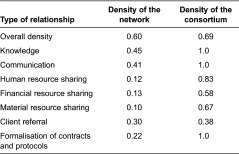
Density of relationships in the traumatic brain injury care network

**Table 2.**
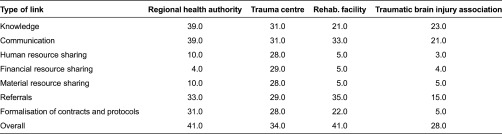
Centrality of consortium organisation members

**Table 3.**
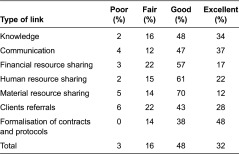
Quality of the links between organisation members of the network

## Appendix 1: Study methodological considerations

**Network boundaries**: Knoke describes the issue of specifying network boundaries in the following terms: ‘Where does a researcher set the limit when collecting data on social relations that, in reality, may have no obvious limits?’ [18] (p. 15) The decision about which individuals or organisations should be considered members of the network is a critical one, equivalent to determining the population of a study [18,27]. Indeed, the omission of an important network member or the arbitrary delineation of network boundaries can lead to inaccurate network measurement [22]. Three types of strategies exist to help researchers set network boundaries: (1) positional strategies, (2) relational strategies and (3) event-based strategies [18]. When researchers use positional strategies, they base their choice of network members upon a given characteristic such as the type of organisation (e.g. hospitals in a given area) or membership in an association. Formal lists often exist to help researchers identify network members on the basis of a given characteristic. In contrast, when they use relational strategies, researchers count on knowledgeable informants (often identified network members) to identify network participants. Informants may be requested to identify these members using free recall or based on a large list provided by the researcher. Snowball sampling is an example of network delineation through a relational strategy. A third and less common strategy to delineate network boundaries involves using participation in one or many events to identify who is a member of the network. The events used must be clearly defined in terms of time and place; they can consist of demonstrations, participation in a meeting or conference, etc.

The study of organisational networks (in contrast to individual networks) poses an additional challenge, because network members are organisations and not individuals. It is impossible to survey ‘organisations’ and social network analysis researchers must determine which individuals within the organisations are knowledgeable enough regarding the network to provide reliable information about their organisation's relationships with other network members. This challenge is also an issue in network activities, where organisation members obviously cannot attend all network meetings or participate in all network actions. Thus, individuals are always asked to speak and act on behalf of their organisation [13], and neither theoreticians nor researchers have proposed solutions to address the potential discrepancies resulting from this.

**Data collection**: In surveys, researchers send questionnaires to the network members asking them to quantitatively describe the relationships they have with each of the other network members. For example, if a network has five members, researchers ask Member 1 to describe the links their organisation has with Members 2, 3, 4 and 5. They also ask Member 2 to describe the links they have with Members 1, 3, 4 and 5, and so on. The link or relationship between two members can be binary (0 = Absent / 1 = Present) or can be weighted according to the link number, frequency, intensity or quality (0 = Absent / 1 = Poor / 2 = Fair / 3 = Excellent). Since many types of relationships can exist at the same time within a network (e.g. communication, resource sharing and referrals), researchers can ask network members to describe as many relationships as required. Other techniques include observation of network members' interactions, or a content analysis of documents such as meeting minutes, papers, databases, etc., that could provide information about a given relationship. Client referral is a good example of relational data that could be gathered in a database. The choice of technique depends upon various practical considerations (e.g. geographical span of the network, time available for data collection, etc.), but more important, it depends on the type of relationships examined. Some relationships are evidenced by the existence of contracts or client referrals between two network members. These are factual and thus can easily be documented by external researchers. Other relationships, such as those involving knowledge or trust, are merely perceived, without tangible evidence of their existence. In the case of perceived relationships, one may be required to directly question network members to obtain the desired data [22].

**Data organisation**. As social network analysis data focus on the relationships between each pair (dyad) of network members, the data are best organised using a matrix. A social network analysis matrix is a table containing as many columns and rows as there are network members. If a network consists of 10 members, the matrix will be 10 x 10. The network member names are used to label the rows and columns of the matrix. The cell located at the intersection of each row and column contains the relational data pertaining to the relationship between the two organisations. Finally, because an organisation cannot have a relationship with itself, the diagonal is left empty.

To facilitate data treatment, a social network analysis matrix can be imported into specialised social network analysis software such as Ucinet [28]. These software programmes contain preprogrammed procedures allowing the data contained within the matrix to be easily organised and further analysed.

The pairs of network members do not always agree concerning their mutual relationship, creating an asymmetrical matrix. Organisation A indicated that it had Fair (‘2’) Client Referrals with Organisation B, but Organisation B reported the same relationship as being Good (‘3’). There are two ways to deal with asymmetrical perceptions of relationships. The first is to use ‘directed’ relationships, which is to analyse separately the perceptions that Organisations A and B have of each other. This solution implies a multiplication of the relationships considered and the analysis becomes more complex since each side of each relationship must be considered. The second way to deal with asymmetrical perceptions is to make them symmetrical. This implies that the researchers always consider relationships (A-B and B-A) as being reciprocal [29]. A special matrix procedure in UCINET software (the ‘Symmetrize’ procedure) can transform a matrix by attributing a maximum, minimum or mean value to the two perceptions of the relationship. In the previous example, A-B and B-A would be attributed the same value ‘3’ if one chooses to keep the maximum value, ‘2’ if one chooses to keep the minimum value, and ‘2.5’ if one chooses to keep the mean value. In addition to simplifying the analysis, the procedure of transforming asymmetrical matrices into symmetrical ones provides a way to deal with missing data. For example, when a network member does not provide answers to the complete survey, the researchers could use the ‘mirror’ data provided by the other members of the dyad to fill in any blank cells. When only one member of the dyad describes a link, the link is labelled as ‘unconfirmed’. Unconfirmed links are considered weaker and less reliable than a ‘confirmed’ link [13] described by both dyad members.

Finally, applying an additional special procedure in UCINET (the ‘Dichotomize’ procedure), a weighted matrix can be made binary. The values ‘1’ to ‘4’ would be assigned the value ‘1’, indicating an existing relationship, while the cells containing a value of ‘0’ (‘No relationship’) would remain the same. The matrix reduction could be useful in simplifying the analysis, or when one wants to count the number of distinct relationships existing in the network by adding the values contained in the different matrices (a procedure also possible in UCINET). In this case, as each existing relationship is given a value of ‘1’, the summative matrix contains the sum of all values contained in a given cell (e.g. cell AB) of the matrix. For example, cell AB of the summated matrix would appear as AB Relationship 1 + AB Relationship 2 + … + AB Relationship I = Sum of AB relationships = Number of relationships existing between the two nodes.

**Data analysis**. Graphs provide useful visual snapshots of networks, enabling an immediate and somewhat intuitive understanding of the network. They allow one to immediately see who has more links, who's related to whom, who is isolated, who should be linked with others and who should not. Specific software (NetDraws, included in UCINET) even allows variations in the shape, colour and weight of the nodes as well as variations in the ties used to reflect various characteristics. The characteristics shown could be based on network measures (discussed below) or on descriptive characteristics (such as genre or type of participants, number or quality of links, and so on) included in the matrix by researchers.

## Appendix 2: Definitions of the relationships included in the questionnaire

**Knowledge**: awareness of the existence of the other organisation

**Communication**: whether they, or someone in their organisation, had spoken to a representative or employee from the other organisation

**Human resource sharing**: whether the organisations shared one or more employees

**Financial resource sharing**: whether the organisations shared and managed a common budget

**Material resource sharing**: whether the organisations shared work space(s), technical devices or material

**Client referrals**: whether the organisations transferred clients to each other

**Formalisation of contracts and protocols**: whether joint formalised procedures and documents existed
